# Reconstruction of the Abdominal Vagus Nerve Using Sural Nerve Grafts in Canine Models

**DOI:** 10.1371/journal.pone.0058903

**Published:** 2013-03-29

**Authors:** Jingbo Liu, Jun Wang, Fen Luo, Zhiming Wang, Yin Wang

**Affiliations:** 1 Department of Hand Surgery, Huashan Hospital, Fudan University, Shanghai, China; 2 Key Laboratory of Hand Reconstruction, Ministry of Health, Shanghai, China; 3 Key Laboratory of Peripheral Nerve and Microsurgery, Shanghai, China; 4 Department of General Surgery, Huashan Hospital, Fudan University, Shanghai, China; 5 Department of Neuropathology, Huashan Hospital, Fudan University, Shanghai, China; Emory University, Georgia Institute of Technology, United States of America

## Abstract

**Background:**

Recently, vagus nerve preservation or reconstruction of vagus has received increasing attention. The present study aimed to investigate the feasibility of reconstructing the severed vagal trunk using an autologous sural nerve graft.

**Methods:**

Ten adult Beagle dogs were randomly assigned to two groups of five, the nerve grafting group (TG) and the vagal resection group (VG). The gastric secretion and emptying functions in both groups were assessed using Hollander insulin and acetaminophen tests before surgery and three months after surgery. All dogs underwent laparotomy under general anesthesia. In TG group, latency and conduction velocity of the action potential in a vagal trunk were measured, and then nerves of 4 cm long were cut from the abdominal anterior and posterior vagal trunks. Two segments of autologous sural nerve were collected for performing end-to-end anastomoses with the cut ends of vagal trunk (8–0 nylon suture, 3 sutures for each anastomosis). Dogs in VG group only underwent partial resections of the anterior and posterior vagal trunks. Laparotomy was performed in dogs of TG group, and latency and conduction velocity of the action potential in their vagal trunks were measured. The grafted nerve segment was removed, and stained with anti-neurofilament protein and toluidine blue.

**Results:**

Latency of the action potential in the vagal trunk was longer after surgery than before surgery in TG group, while the conduction velocity was lower after surgery. The gastric secretion and emptying functions were weaker after surgery in dogs of both groups, but in TG group they were significantly better than in VG group. Anti-neurofilament protein staining and toluidine blue staining showed there were nerve fibers crossing the anastomosis of the vagus and sural nerves in dogs of TG group.

**Conclusion:**

Reconstruction of the vagus nerve using the sural nerve is technically feasible.

## Introduction

Radical resection of gastric cancer is currently the major procedure for treating of gastric cancer. Generally, the anterior and posterior vagal trunks should be cut off for complete dissection of the first, second and third groups of lymph nodes in the stomach.

Owing to fact that the hepatobiliary and abdominal cavity branches are cut at the same, removal of the anterior and posterior vagal trunks leads to a significant increase in the incidence of post-surgical gallstones [1], [2], affects the vagal innervation of the small intestine, parts of the colon, and the pancreas, and affects the post-surgical digestive and absorptive functions of patients. Recently, with increases in the detection of early-stage gastric cancer and improvements in the techniques of radical operation, the overall 5-year survival rate in gastric cancer cases has improved greatly. Radical resection of gastric cancer focuses not only on completion of the resection, but also on preservation of the functions of organs as much as possible. Many experimental and clinical studies have found that radical resection of gastric cancer with vagus nerve preservation reduced the occurrence of post-surgical gallstones, increased gastrointestinal motility and gastric emptying ability, reduced the occurrence of post-surgical gastro-esophageal reflux, accelerated post-operative body weight recovery, and significantly improved post-surgical quality of life [3]–[5].

In some countries radical resection of gastric cancer with vagus nerve preservation has been performed, which mostly preserves the hepatobiliary and abdominal cavity branches of the nerve. Some are carried out under laparotomy, others under laparoscopy. The procedure has become a very active topic of research of surgical techniques.

However, whichever technique of vagus nerve preservation is employed in radical resection of gastric cancer, its use is mostly limited to early-stage disease. This may be attributed to the following: (1) Progression-stage gastric cancer has many perigastric metastatic lymph nodes, and preservation of the vagus nerve may affect the removal of the nodes. (2) As progression-stage gastric cancer may directly invade the vagus nerve, preservation of the nerve may lead to incomplete removal of the tumor. Therefore, in order to completely remove the first group of lymph nodes and preserve the function of the hepatobiliary branch of the vagus nerve and the function of pylorus, the removal of the No.1 lymph node with resection of parts of the hepatobiliary branch was performed, followed by *in situ* anastomosis and reconstruction, which achieved good clinical outcomes [6], [7]. However, that method cannot ensure removal of the No.3 and No.7 lymph nodes, unless parts of the vagus nerve are removed.

The best known situation in which removal of nerves during radical treatment of a malignant tumor leads to impairment of function is that of prostate cancer. In radical resection of prostate cancer, it is often necessary to remove the cavernous nerve, which causes post-operative erectile dysfunction [8]. Currently, two methods are used to improve post-operative erectile function: (1) unilateral nerve-sparing radical prostatectomy; and (2) unilateral nerve-sparing radical prostatectomy plus a sural nerve graft [5]. It has been reported that post-operative erectile function recovers significantly in patients who have undergone nerve preservation or nerve grafting [9]–[11].

Based on the success of partial resection of the vagus nerve followed by re-anastomosis and reconstruction for progression-stage gastric cancer (T2, T3) [6], [7], the widespread use of cutaneous nerve grafts for repairing nerve defects in plastic surgery, and the fact that the sural nerve is employed to repair parasympathetic nerves in urological surgery, we hypothesized that resection of the vagus followed by re-anastomosis of the anterior and posterior vagal trunks and the hepatobiliary and abdominal cavity branches of the vagus nerve with sections of autologous sural nerve would preserve nerve function and improve quality of life by ensuring complete removal of the No.3 and No.7 lymph nodes.

In order to test this hypothesis, the present study explored the surgical techniques for repairing the hepatobiliary and abdominal cavity branches of the vagus nerve using the sural nerve, seeking experimental evidence for nerve regeneration after graft repair, and for recovery of secretary function of the gastrointestinal tract.

## Materials and Methods

### Materials

Ten adult Beagle dogs, each weighing 9–12 kg, were purchased from the Laboratory Animal Center of School of Agriculture and Biology, Shanghai Jiantong University. The action potential of the nerve trunk was measured using the Dantec Keypoint® electromyography (EMG)/evoked potentials (EP) system (Alpine Biomed, Denmark). Chitosan was purchased from Shanghai Qisheng Biological Preparation Co., Ltd. (Shanghai, China). Monoclonal mouse anti-human neuroflilament protein (NF) was supplied by Dako (clone number: 2F11). The animals were randomly assigned to two groups of five, the nerve grafting group (TG) and the vagal resection group (VG). The protocols in this study were approved by institutional review board and the Animal Care and Use Committee of Huashan Hospital, Fudan University.

### Detection of action potential of sural nerve graft and normal vagal trunk

**Figure 1:**
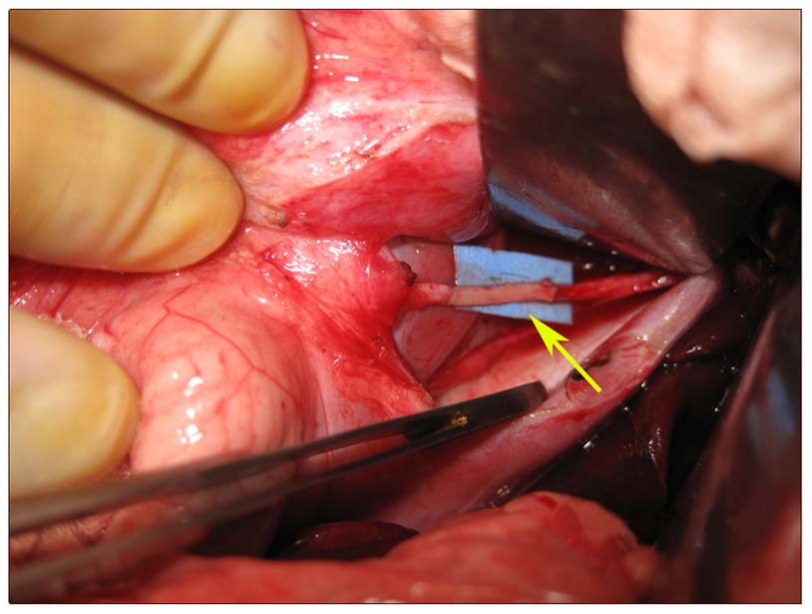
Anastomosis of the vagus nerve with the grafted segment of the sural nerve. The arrow indicates the grafted segment.

The dogs in the TG group were injected with phenobarbital 25 mg/kg for induction of general anesthesia. Median laparotomy was performed in the middle and upper abdomen; the hepatogastric ligament and the left diaphragmatic angle were opened; and the abdominal esophagus was exposed to gain access to the anterior and posterior vagal trunks. A trunk of about 4.0 cm long was isolated, and stimulation and recording electrodes were placed on both sides 4.0 cm apart. The latency and conduction velocity of the action potential were measured and recorded. Segments of the anterior and posterior trunks were cut off 3 cm anterior to the hepatobiliary branch of the anterior vagal trunk, and the abdominal cavity branch of the posterior vagal trunk, each about 3.5 cm long. Two segments of sural nerve (4 cm) in the left lower limbs of dogs in the TG group were resected. End-to-end anastomosis was performed between the broken ends of the anterior and posterior vagal trunks and the epineurium of the sural nerve, using three sutures of 8–0 proline for each anastomosis (Fig. 1). The anastomosis site was sprayed with chitosan to prevent post-operative adhesion of the site to surrounding tissues.

Anesthesia and nerve isolation in dogs of the VG group were similar to those in the TG group, while the action potential was not measured. After removing parts of the vagal trunk (3.5 cm long), the cut part of the nerve was not anastomosed. When the stomach loses vagal innervation, the emptying and secretion functions are significantly affected. Whether the sural nerve graft led to recovery of the vagal innervation of the stomach could be studied for comparison with the TG group. All dogs received intravenous antibiotics. After two days of surgery, the dogs were given a liquid diet, after which a solid diet was provided.

### Assessment of gastric secretion and emptying function

Gastric secretion and emptying function were assessed in dogs of the VG and TG group before surgery and three months after surgery. For gastric secretion function the Hollander insulin test [9] was used. The gastric tube was implanted in all dogs under general anesthesia after fasting for 24 h. After the gastric juice had drained out, it was collected every 10 min for a total of 6 times Dogs were then injected intravenously with insulin 0.2 U/kg, and the gastric juice was collected a further 6 times, again every 10 min. The acidity of gastric acid was titrated with 0.01 M NaOH. The maximal acid output (MAO) was calculated using the following formula:
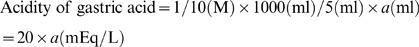


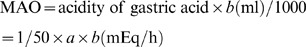
Where *a* indicates the amount of NaOH used for 5 ml of gastric acid titration solution with a pH value of 7, and *b* indicates the amount of gastric juice for 1 h.

Gastric emptying function was assessed using the acetaminophen test. After fasting for 24 h, the dogs were given 20 g bread and 200 ml milk containing acetaminophen at a dose of 0.025 g/kg body weight. After 45 min, blood samples were collected, and the acetaminophen concentration in blood was determined using high performance liquid chromatography (HPLC) [12].

### Collection of the grafted nerve samples and detection of action potential in the nerve trunk

Laparotomy was performed in dogs of the TG group; the grafted segment of the vagus nerve was isolated; and the electrode was implanted into the lateral sides of the two anastomosis sites to record the action potential in the nerve trunk again. The grafted nerve and the anastomosis site were removed for examination. After surgery, the dogs in the TG group were sacrificed by injection with air. The dogs in the VG group did not undergo any other treatment, and were not sacrificed. All dogs in the TG group survived till the second surgery, while one dog in the VG group died of diarrhea one month after grafting.

### Histology of the grafted nerve

Three grafted nerve samples were collected for examination in the Department of Neuropathology, Huashan Hospital Affiliated to Fudan University. Longitudinal sections were cut in the proximal and distal anastomosis sites, which were then stained with anti-NF. Two samples were collected for examination in the Section of Electron Microscopy, Shanghai Medical College of Fudan University. Semi-thin transverse sections were cut in the upper part of the proximal anastomosis site, lower part of the distal anastomosis site, and the grafted nerve, and then stained with toluidine blue.

### Statistical analysis

All data were expressed as mean ± standard deviation (SD), and all statistical analyses were performed using the statistical software SPSS version 13.0 (SPSS, CA, USA). Differences between indices before and after surgery in the same group were tested for statistical significance using paired Student's *t*-test, while Student's *t*-test was used to compare different groups.

## Results

### Nerve grafting

Fig. 1 shows the anterior vagal trunk. A segment about 4 cm long was removed. The nerve between the two anastomosis sites is the grafted sural nerve.

### Determination of action potential of the nerve trunk

**Figure 2:**
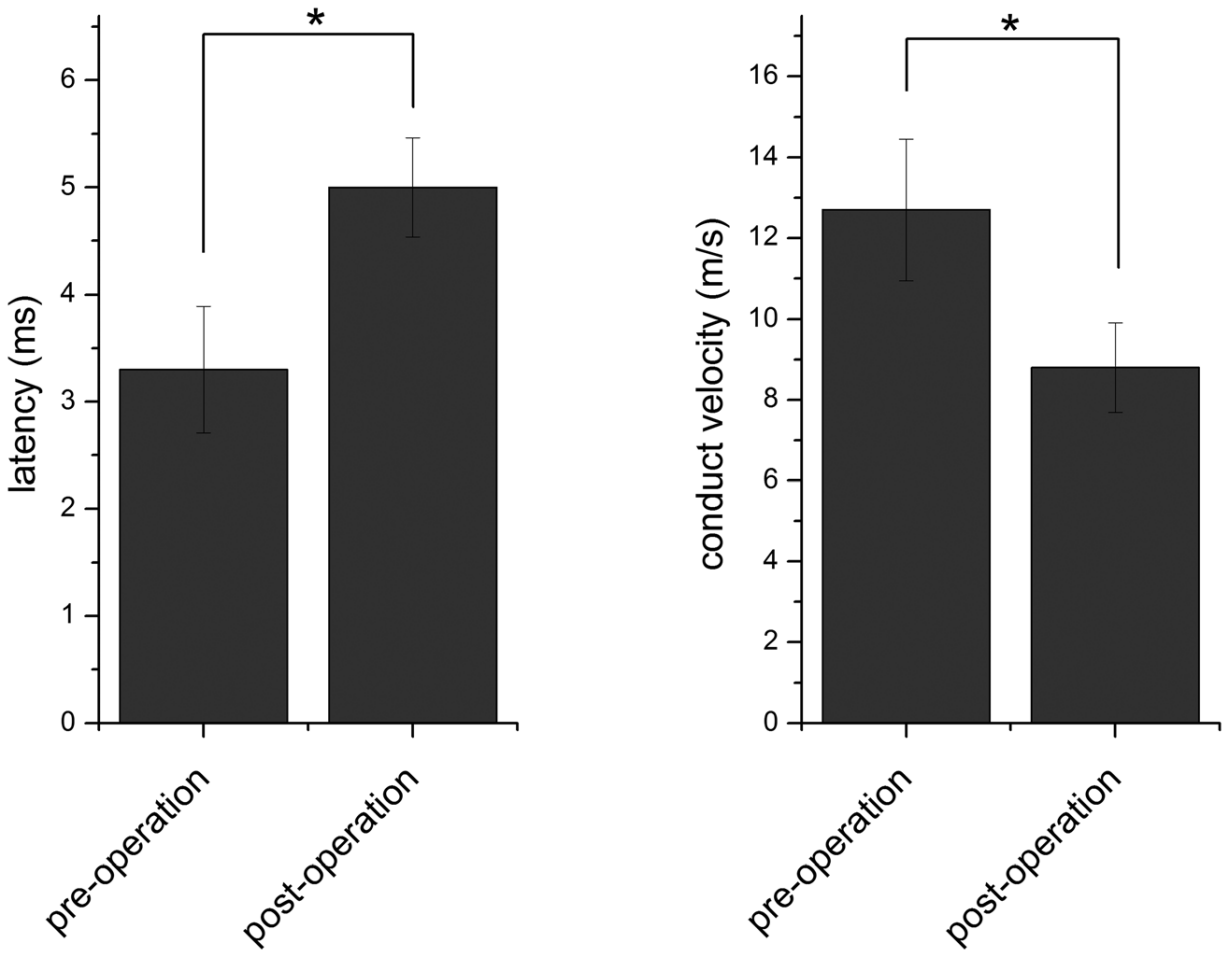
Changes in the latency and conduction velocity of the action potential in the vagal trunk of dogs in the TG group before and after surgery. * The difference before and after surgery was statistically significant (*P*<0.05).

The mean latency in the TG group after surgery was 5.0±0.5 ms, compared with 3.3±0.6 ms before surgery (*P*<0.05) (Fig. 2). The mean conduction velocity was 12.7±1.8 m/s before surgery, and 8.8±1.1 m/s after surgery (*P*<0.05). These results suggested that the grafted sural nerve retained some conduction function, although it was reduced.

### Histomorphology of the grafted nerve

**Figure 3:**
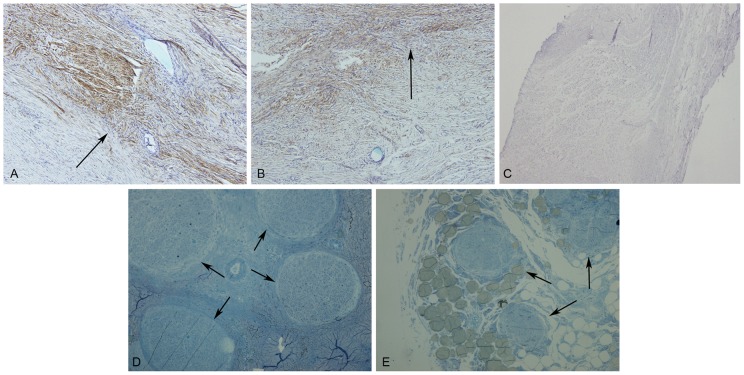
The anastomosis sites of the grafted nerves. A. The anastomosis site on the proximal grafted nerve (NF, ×40). The arrow indicates the anastomosis site. B. The anastomosis site on the distal grafted nerve (NF, ×40). The arrow indicates the anastomosis site. C. Negative control. D. The transverse section of the grafted nerve on the proximal grafted nerve. The arrow indicates the nerve fiber bundle. E. Transverse section of the vagus nerve on the distal grafted nerve. The arrow indicates the nerve fiber bundle.

The NF staining of the grafted nerve with the anastomosis site is shown in Fig. 3A and B. The brown part of the figures indicates NF-positive nerve fibers. NF staining was positive on both sides of the anastomosis site. Toluidine blue staining of the semi-thin sections is shown in Fig. 3D and E. The blue dots in the figures indicate the cross-sections of the nerve fibers. It was found that there were nerve fibers crossing the anterior and posterior parts of the anastomosis site in the distal grafted nerve. After nerve injury, Wallerian degeneration occurred in the distal injured nerve within 2 weeks, and the axon disintegrated and was assimilated. If no regeneration of the grafted nerve occurred, the NF staining in the nerve of the distal anastomosis site appeared negative. Therefore, NF staining revealed that the regenerated nerve fibers had crossed the anastomosis site.

### Gastric emptying and secretion function

**Figure 4:**
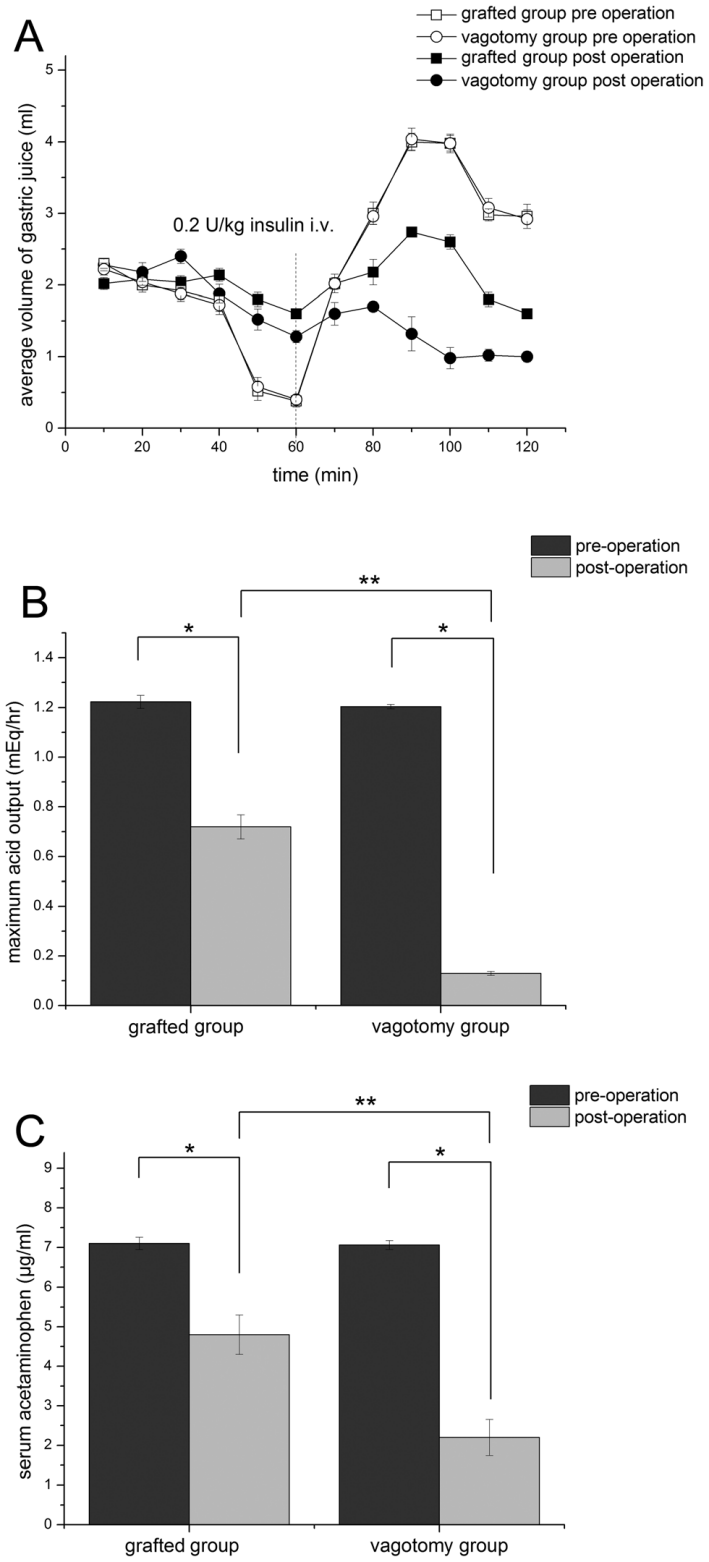
Gastric emptying and secretion function. A. Pre- and post-operation average volume of gastric juice secretion in vagotomy and grafted group. The vertical line indicates the i.v. application of insulin. B. Pre- and post-operation maximum acid output in vagotomy and grafted group. C. Pre- and post-operation serum acetaminophen in vagotomy and grafted group. * shows there are significant difference between pre- and post-operation (*P*<0.05). ** shows there are significant difference between vagotomy and grafted group (*P*<0.05).

Fig. 4A shows the average amount of gastric juice collected at each time point before and after surgery in the TG and VG groups. Fig. 4B shows MAO before and after surgery in the two groups. The difference was significantly smaller in the TG group than in the VG group, demonstrating that the recovery of gastric secretion function in the TG group was greater. Fig. 4C shows the results of the acetaminophen tests before and after surgery. In both groups serum acetaminophen concentration was significantly lower after surgery than before surgery. However, the difference in concentration before and after surgery in the TG group was significantly smaller than that in the VG group (*P*<0.05), indicating that the recovery of gastric emptying function was greater in the TG group than in the VG group.

## Discussion

Our study has established the feasibility of recovering vagus nerve function by using a graft taken from the sural nerve. Two to three months after the sural nerve was grafted, both nerve staining and the detection of an action potential proved that the grafted nerve crossed the anastomosis site. Determination of gastric secretion and emptying function also showed that the neural innervation of stomach had partially recovered. Although the gastric emptying and secretion functions in the TG group were weaker than in the normal group, they were superior to those in the VG group.

When designing the experiment, there were two key questions: (1) Which autologous nerve should be selected as the graft donor? Or alternatively, should an artificial nerve donor be used? (2) Should both the hepatobiliary and abdominal cavity branches of the vagus nerve be removed and reconstructed, or should one be preserved while the other is reconstructed?

In this study, autologous sural nerve was used for nerve repair, which is widely used in peripheral nerve repair surgery. For instance, in repairing the cavernous nerve with the sural in urological surgery [13]. The problem of prostate cancer treatment is similar to that of treating gastric cancer. To ensure radical resection of tumors, the nerve may be cut, which leads to erectile dysfunction. Sural nerve grafting has been shown to be beneficial to patients with sexual dysfunction after prostate cancer surgery [13]. It has been reported that repair of the cavernous nerve using sural nerve grafts leads to 40%–60% recovery of erectile function [9], [11], [14]. Both the cavernous and vagus nerves are parasympathetic. Considering that sural nerve grafting can restore the function of the cavernous nerve, it was hoped that it could be used to restore vagus nerve function also. In addition, the sural nerve is easy to collect; the wound in the donor site is small; and its diameter is similar to that of the vagus nerve. Compared with an artificial nerve graft [15], a sural nerve graft provides not only a holder for nerve growth, but also nerve growth factor, which is why it was selected for the present study.

In this study, the two branches of the vagus nerve, hepatobiliary and abdominal cavity branches, were resected together and then reconstructed, which can be explained by two reasons. Firstly, since gastric cancer has a characteristic of submucosal diffusion and growth, and hepatobiliary and abdominal cavity branches both belong to the second station of nodes, these two branches then were generally both removed in a D2 radical resection in the progressive stage of gastric cancer. Secondly, In the present study, if we preserved a branch of the vagus nerve, it was difficult to prove that recovery of the function of the gastrointestinal tract was attributable to nerve reconstruction or nerve preservation, which was the second basis for resection and reconstruction of the hepatobiliary and abdominal cavity branches.

Our study has established the feasibility of improving the recovery of the function of the gastrointestinal tract using nerve grafting during radical resection of progression-stage of gastric cancer. Further studies are merited to investigate the possibility of replacing the autologous nerve with an artificial nerve graft plus nerve growth factor to overcome the problem of numbness and pain in the nerve donor region.
